# An Environmentally Benign Protocol for Aqueous Synthesis of Tetrahydrobenzo[*b*]Pyrans Catalyzed by Cost-Effective Ionic Liquid

**DOI:** 10.3390/ijms15046897

**Published:** 2014-04-22

**Authors:** Huanan Hu, Fangli Qiu, Anguo Ying, Jianguo Yang, Haiping Meng

**Affiliations:** 1 Institute of Applied Chemistry, Taizhou University, Taizhou 318000, China; E-Mails: hhn12192000@126.com (H.H.); lhqiu123@163.com (F.Q.); 2 School of Pharmaceutical and Chemical Engineering, Taizhou University, Taizhou 318000, China; E-Mail: mhp@tzc.edu.cn

**Keywords:** pyran, choline hydroxide, ionic liquid, cost-effectiveness, biodegradability, recyclability

## Abstract

A mild, efficient, and environmentally benign protocol for the synthesis of tetrahydrobenzo[*b*]pyran derivatives in the presence of readily accessible, biodegradable, and choline hydroxide based ionic liquid as catalyst has been established. The key features of the reported methodology include good to excellent yields of desired products, simple work-up procedure and good recyclability of catalysts, which may be a practical alternative to the existing conventional processes for the preparation of 4-*H* pyrans to cater to the requirements of academia as well as industry.

## 1. Introduction

As an important class of oxygen-containing heterocycles, tetrahydrobenzo[*b*]pyrans are widely employed as potential biodegradable agrochemicals [1], photoactive materials [2], cosmetics and pigments [3]. Because of the inherent reactivity of the pyran ring, 4-*H* pyrans are versatile synthons, which can be easily converted into pyridine compounds as pharmacologically important calcium antagonists [4,5]. Tetrahedronhydro[*b*]pyran compounds themselves have a broad spectrum of biological properties [6,7], such as spasmolytic, anticancer, diuretic, anticoagulant, and antiancaphylactia activities [8,9]. They can also be used as cognitive enhancers not only for the treatment of neurodegenerative disease, for example, Parkinson’s disease, Huntington’s disease, Alzheimer’s disease, amyotrophic lateral sclerosis, AIDS associated dementia and Down syndrome but also for the treatment of schizophrenia and myoclonus [10]. Due to the versatile utilization of the pyran derivatives in the field of organic synthesis as well as in medicinal chemistry, many researchers have been encouraged to develop highly efficient procedures for the preparation of these kinds of compounds.

Multi-components reactions (MCRs), an important subclass of tandem reactions, are one-pot processes in which complicated molecules, especially various heterocycles with biological activities, can be produced in a very fast, efficient, and economic manner without the isolation of any intermediate [11–13]. Hence, considering the fascinating advantages of MCRs, some methodologies for the synthesis of tetrahedrondydro[*b*]pyrans via three components one-pot reactions catalyzed by 1,4-diazabicyclo[2.2.2]octane [14], *N*-methylimidazole [15], tetra-methyl ammonium hydroxide [16], (NH_4_)_2_HPO_4_ [17] K_3_PO_4_ [18], ZnO-beta zeolite [19], nanosized Ce_1_MgXZr_1−_*_x_*O_2_ [20], Ru(II) complex [21], Na_2_SeO_4_ [22], *S*-proline [23], l-proline [24], 1,8-diazabicyclo[5.4.0]undec-7-ene [25], sulfonic acid functionalized silica [26], phenylboronic acid [27], caro’s acid-silica gel [28], and cerium(III) chloride [29] have been reported. In addition, procedures of catalyst-free and 2,2,2-trifluoroethanol as reaction medium [30], microwave and ultrasonic irradiation [9,31,32] could also give satisfactory yields of pyrans. Each of the above methods has its own merits, while some are plagued by one or more limitations of tedious work-up, poor product yields, long reaction times, effluent pollution, unavailability of the catalyst and vast employment of organic solvents, which is significantly harmful to environment. Consequently, gaps remain in terms of the search for alternative methods for synthesis of 4-*H* pyrans which are both high efficiency and environmentally friendly.

**Scheme 1.**
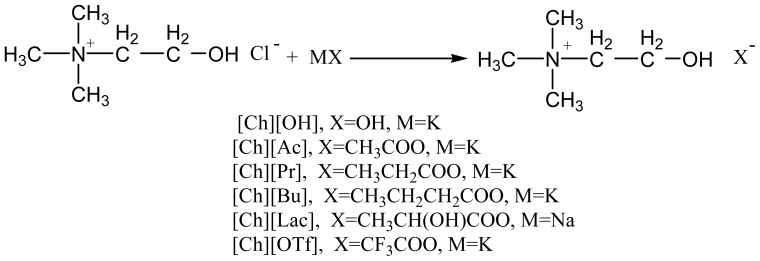
Preparation of choline hydroxide based ionic liquids.

Ionic quids (ILs) with the unique properties of low volatility, good and tunable solubility, non-flammability, and excellent recyclability are receiving considerable global attention and used extensively in organic synthesis as green reaction media or designable catalysts [33–40]. Thus, some task-specific ionic liquids were successfully introduced as catalysts for the preparation of the desired tetrahydrobenzo[*b*]pyran derivates [41–44]. However, disadvantages associated with high price, a little toxicity and difficulty of biodegradability, to some extent, limit these methodologies at the industrial scale. Choline chloride (ChCl), a member of the vitamin B family, is a very cheap, commercially available, biodegradable and non-toxic quarternary ammonium salt, which can be simply produced by gas phase reaction or directly extracted from biomass [45,46]. Compared to the intensive studies of ChCl-derived deep eutectic solvents as catalysts for organic transformations [45,46] the research with regard to the application of ChCl-based ionic liquids to organic synthesis is very rare [47]. As part of our ongoing pursuit for the establishment of “green organic synthesis” [48–56], we herein prepared a type of non-toxic, cheap and biodegradable ChCl-derived ionic liquids and present their usage as catalysts for the synthesis of 4*H*-benzo[b]pyrans via MCRs of aldehydes, methylene active compounds and 5,5-dimethyl-1,3-cyclohexanedione in aqueous medium. These type of ionic liquids can be readily prepared through anion exchange reactions (Scheme 1) and they are all liquids at room temperature (25 °C).

## 2. Results and Discussion

Initially, the reaction of *p*-anisaldehyde, malononitrile and dimedone was selected as model for the optimization of reaction conditions. As shown in Table 1, [bmim]BF_4_ and [bmim]PF_6_, two traditional ionic liquids frequently used as reaction media for organic transformations, gave significantly lower yields of products (Table 1, entries 1–2). Moreover, new DBU derived ILs were found to be less suitable as catalysts for the preparation of the pyran product while they exhibited excellent catalytic activities for aza-Michael addition [48–50] and Knoevenagel condensation [48–56] (Table 1, entries 3 and 4). We then turn our attention to the cost-effective and biodegradable ILs, [Ch][X]. Among the ILs [Ch][X] examined as catalysts for the model reaction, [Ch][OH] could efficiently catalyze to form tetrahydro[*b*]pyrans with excellent yield in short time, and the remained ILs, including [Ch][Ac], [Ch][Lac], [Ch][Pr], and [Ch][OTf] exhibited relatively lower catalytic activities (Table 1, entries 5–11). For comparison, [bmim]OH, a basic ionic liquid widely used in organic catalysis, gave slightly lower yield (86%). Combination of ILs’s accessibility, availability and their catalytic efficiency, [Ch][OH] was selected as the best catalyst for the preparation of the functionalized pyrans. Upon investigating the influence of the amount of catalyst on the reaction, it was found that 10 mol % of [Ch][OH] was sufficient to promote the one-pot reaction (Table 1, entries 12–15). Moreover, a brief screening of solvents showed that the much better results in terms of both reaction rate and yield were observed with water as reaction medium than those performed in organic solvents (Table 1, entries 13 and 16–19).

To extend the scope and demonstrate the generality of the present method, we explored the reaction of various aromatic aldehydes with malononitrile and dimedone under the optimized reaction conditions to furnish respective substituted pyrans and the results are demonstrated at Table 2. To our pleasure, the reactions proceeded smoothly and good to excellent yields of desired products were obtained within several hours. The electronic nature of substituents on the aromatic ring has some effects on the transformation. The aromatic aldehydes bearing electron-donating groups such as MeO, OH, 3,4-2(Me) reacted much slower with malononitrile and dimedone than other aromatic aldehydes substituted with NO_2_, CN, F, Cl, Br (Table 2, entries 1–10). The heterocyclic aldehydes such as 2-thienyl and furyl aldedydes were also demonstrated to be efficient reagents for this reaction (Table 2, entries 12 and 14). However, when pyridine and 2-naphthyl aldehydes were subjected to the procedures, only intermediates-Knoevenagel condensation products were detected (Table 2, entries 11 and 13). In addition, the reaction of *trans*-cinnamaldehyde with malononitrile and dimedone was performed under the same conditions and some lower yield (69%) was observed (Table 2, entry 14). It is noteworthy to mention that aliphatic aldehydes were suitable for the condensation achieving good product yields (Table 2, entries 16 and 17).

**Scheme 2.**
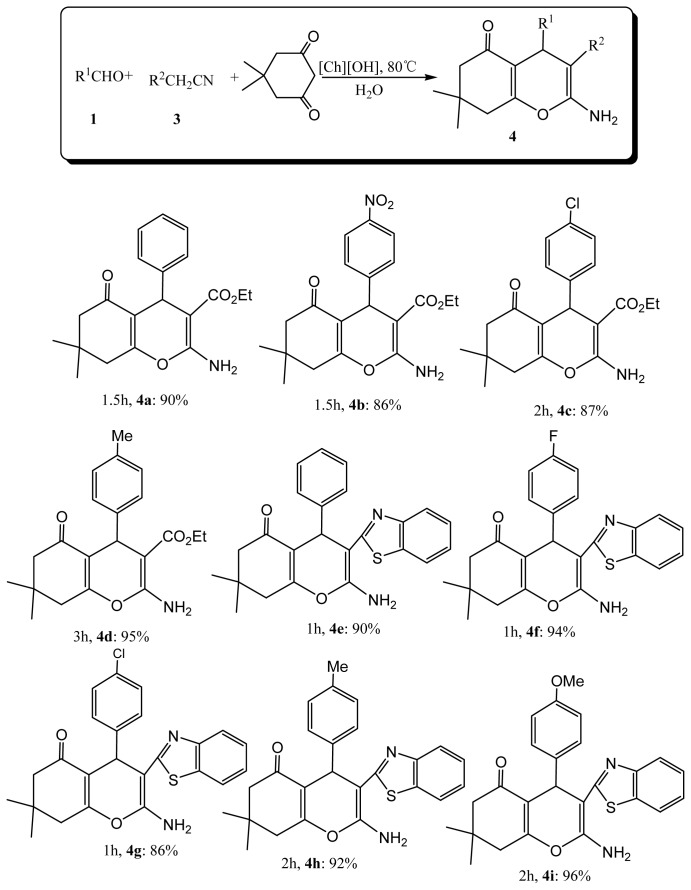
[Ch][OH] promoted one-pot synthesis of 4*H*-benzo[*b*]pyrans via the reaction of aromatic aldehydes, cyanoacetate or 2-(benzo[d]thiazol-2-yl)acetonitrile, and dimedone.

Encouraged by the exciting results from the reactions of malononitrile, we decided to further extend the substrate scope and two CN substituted active methylene compounds, cyanoacetate and 2-(benzo[d]thiazol-2-yl)acetonitrile were tested (Scheme 2). As outlined in Scheme 2, the novel ionic liquid [Ch][OH] as catalyst is suitable for cyanoacetate as well as 2-(benzo[d]thiazol-2-yl)acetonitrile and can afford the corresponding heterocyclic compounds in good yields (Scheme 2, **4a**–**i**).

**Figure 1.**
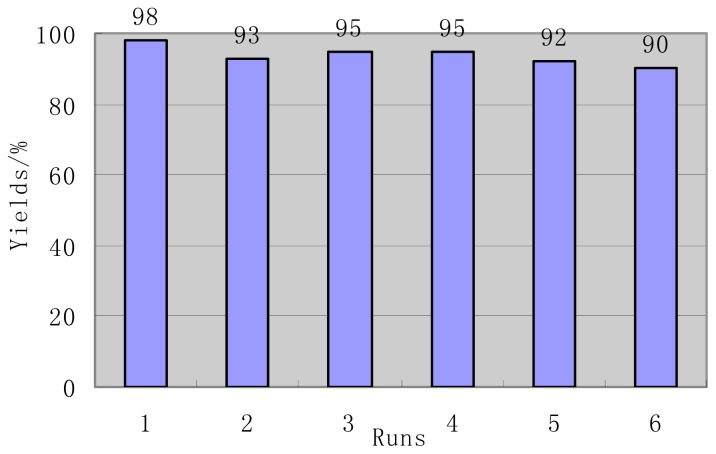
Reuse of ionic liquid for three components condensation of *p*-anisaldehyde (100 mmol), malononitrile and dimedone in the presence of ionic liquid [Ch]][OH] in aqueous solution.

In order to demonstrate the industrial applicability of this methodology, the aqueous one-pot synthesis of 4*H*-benzo[*b*]pyrans via the reaction of *p*-anisaldehyde, malononitrile, dimedone catalyzed by [Ch]][OH] was carried out on a larger scale (100 mmol). The reaction was completed in 2 h. The excellent yield of 98% for the conjugate product was obtained. On the same scale, the recyclability of the catalytic system was investigated using the same reaction as model reaction. Upon the completion of the reaction, the product was isolated by filtration while the filtrate was dried to remove water at 80 °C under vacuum. The recovered ionic liquid was reused in subsequent reactions. As shown in Figure 1, the ionic liquid [Ch][OH] can be recycled 5 times without considerable decrease of activity and the used ionic liquid remained intact (^1^H NMR).

**Scheme 3.**
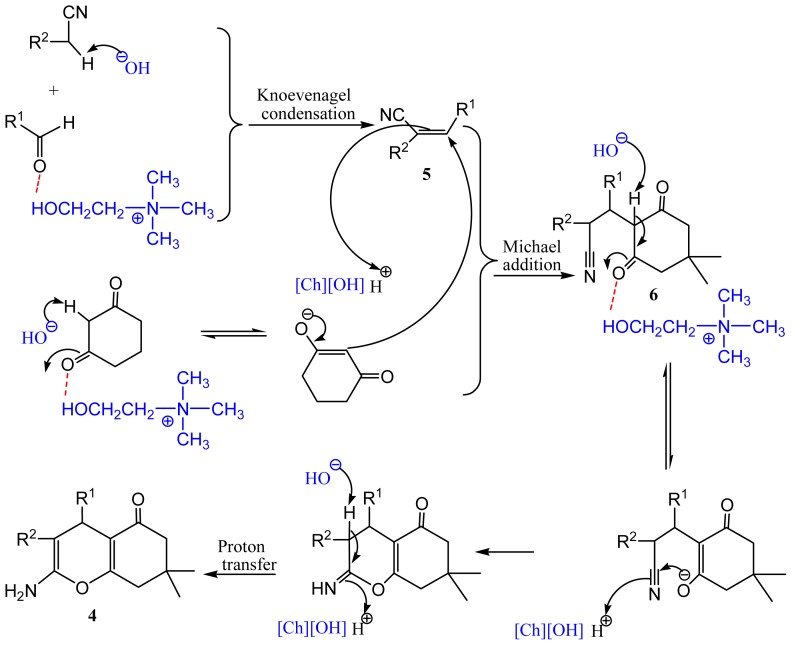
The proposed mechanism for [Ch][OH] promoted synthesis of tetrahydrobenzo[*b*]pyrans.

A plausible mechanism for the reactions is illustrated in Scheme 3. Due to the strong basicity of hydroxyl anion of the IL (see Table S1) and hydrogen bond formed between hydroxyl group of side chain of Ch and carboxyl moiety of aldehydes, the dual activation of methylene ingredients and aldehydes facilitate the formation of a Knoevenagel condensation product **5**. Dimedone can be easily converted to its enolic form in the presence of [Ch][OH] that could readily react with acrylonitrile **5**, affording the Michael addition product **6**, followed by tautomerism, intramolecular *O*-cyclization and proton transfer reactions under dual activities of the IL to give the desired product **4**. ^13^C NMR data of benzaldehyde and the mixture of IL [Ch][OH] and benzaldehyde also provided the catalytic role of the hydrogen bond (see Figures S1 and S2).

## 3. Experimental Section

### 3.1. Materials and General Methods

NMR spectra were recorded Bruker Advance DPX 400 MHz spectrometer (Bruker BioSpin Corporation, Fällanden, Switzerland) with chemical shift values (δ) in parts per million, relative to the internal standard of tetramethylsilane (TMS). Melting points were determined using YRT-3 apparatus (Reliant Instument, Tianjin, China) and were not corrected. All chemical were purchased from Aladdin, Aldrich or Fluka (Buches SG, Switzerland). All reactions were monitored by thin layer chromatography (TLC).

### 3.2. General Procedure for the Synthesis of the ChCl-Based Ionic Liquids

A mixture of ChCl (20 mmol), MX (20 mmol) and methanol (25 mL) was stirred at reflux for 12 h. Then methanol was evaporated at 60 °C in high vacuum until the weight of the residue remained constant. The resulting liquids were the [Ch]X. The ionic liquids were characterized by ^1^H NMR, ^13^C NMR (Bruker BioSpin Corporation, Fällanden, Switzerland).

**[Ch][OH]** ^1^H NMR (400 M, D_2_O): δ4.05 (m, 2H), 3.49 (t, 2H, *J* = 5.2 Hz), 3.20 (s, 9H); ^13^C NMR (100 M, D_2_O): δ 67.86, 55.73, 53.91, 53.84, 48.92.

**[Ch][Ac]** ^1^H NMR (400 M, D_2_O): δ3.98 (m, 2H), 3.44 (t, 2H, *J* = 4.8 Hz), 3.13 (s, 9H), 1.83 (s, 3H); ^13^C NMR (100 M, D_2_O): δ 180.90, 67.42, 55.57, 53.84, 23.46.

**[Ch][Pr]** ^1^H NMR (400 M, D_2_O): δ4.03 (m, 2H), 3.48 (t, 2H, *J* = 5.2 Hz), 3.16 (s, 9H), 2.12 (t, 2H, *J* = 7.2 Hz), 0.86 (t, 3 H, *J* = 7.2 Hz); ^13^C NMR (100 M, D_2_O): δ 183.73, 67.49, 55.68, 54.02, 53.97, 53.94, 39.73, 13.46.

**[Ch][Bu]** ^1^H NMR (400 M, D_2_O): δ 3.98 (m, 2H), 3.44 (t, 2H, *J* = 4.8 Hz), 3.13 (s, 9H), 2.08 (t, 2H, *J* = 7.2 Hz), 1.48 (m, 2H), 0.82 (t, 3H, *J* = 7.2 Hz); ^13^C NMR (100 M, D_2_O): δ 183.78, 67.21, 55.58, 53.90, 53.97, .53.86, 39.67, 19.44, 13.43.

**[Ch][Lac]** ^1^H NMR (400 M, D_2_O): δ3.82 (m, 3H), 3.26 (t, 2H, *J* = 4.8 Hz), 2.98 (s, 9H), 1.07 (d, 3H, *J* = 6.8 Hz), 0.86 (t, 3H, *J* = 7.2 Hz); ^13^C NMR (100 M, D_2_O): δ 182.40, 68.53, 67.47, 55.67, 53.96, 53.93, 53.89, 20.22.

**[Ch][OTf]** ^1^H NMR (400 M, D_2_O): δ 4.02 (m, 2H), 3.48 (t, 2H, *J* = 4.8 Hz), 3.16 (s, 9H); ^13^C NMR (100 M, D_2_O): δ 70.42, 58.61, 56.83.

### 3.3. Typical Procedure for One-Pot Synthesis of Tetrahydrobenzo[b]Pyrans

To a mixture of aromatic aldehyde (5 mmol), methyl active compound (5 mmol), and dimedone (5 mmol) in water (10 mL), [Ch][OH] was added. Upon addition, the reaction was stirred at reflux until the disappearance of starting material by TLC. After the completion of the reaction, the reaction mixture was filtrated to obtain a solid, which was recrystallized in 95% ethanol to give pure product. The ionic liquid was recovered from the remaining filtrate, subsequently remove water in vacuum at 80 °C and reused several times without further purification. The product was characterized by melting point measurement and NMR.

### 3.4. Spectroscopic Data of Typical Products

**2-Amino-3-cyano-4-phenyl-7,7-dimethyl-5-oxo-4*H*-5,6,7,8-tetrahydrobenzo[*b*]pyran** (**2a**): mp 233–235 °C. IR (KBr): 3310, 2966, 2188, 1658, 1372 cm^−1; 1^H NMR (200 MHz, CDCl_3_) (ppm): 1.04 (s, 3H, CH_3_), 1.11 (s, 3H, CH_3_), 2.23 (s, 2H, CH_2_), 2.46 (s, 2H, CH_2_), 4.40 (s, 1H, CH), 4.58 (s, 2H, NH_2_), 7.21–7.30 (m, 5H, Ar-H); ESI-MS: *m*/*z* 294 [M]^+^.

**2-Amino-3-cyano-4-(4-hydroxyphenyl)-7,7-dimethyl-5-oxo-4*H*-5,6,7,8-tetrahydrobenzo[*b*]pyran** (**2h**): mp 205–206 °C. IR (KBr): 3353, 2958, 2170, 1660, 1395, 1218 cm^−1; 1^H NMR (200 MHz, CDCl_3_) (ppm): 1.04 (s, 3H, CH_3_), 1.25 (s, 3H, CH_3_), 2.23 (s, 2H, CH_2_), 2.44 (s, 2H, CH_2_), 4.35 (s, 1H, CH), 4.49 (s, 2H, NH_2_), 6.71–7.27 (m, 4H, Ar-H); ESI-MS: *m*/*z* 333 [M + Na]^+^.

**2-Amino-3-cyano-4-(4-methoxyphenyl)-7,7-dimethyl-5-oxo-4*H*-5,6,7,8-tetrahydrobenzo[*b*]pyran** (**2i**): mp 198–201 °C. IR (KBr): 3356, 2970, 2158, 1656, 1385, 1219 cm^−1; 1^H NMR (200 MHz, CDCl_3_) (ppm): 1.04 (s, 3H, CH_3_), 1.22 (s, 3H, CH_3_), 2.34–2.39 (t, *J* = 10 Hz, 2H, CH_2_), 3.77 (s, 1H, CH), 6.78–7.26 (m, 4H, Ar-H); ESI-MS: *m*/*z* 324 [M + Na]^+^.

**2-Amino-3-(benzo[*d*]thiazol-2-yl)-4-(4-fluorophenyl)-7,7-dimethyl-5-carbonyl-5,6,7,8-4*H*-benzo[*b*] pyran** (**4f**): mp 190–192 °C. IR (KBr): 3325, 1665, 1617, 1482, 1450 cm^−1; 1^H NMR (200 MHz, DMSO-*d*_6_) (ppm): 1.01 (s, 3H, CH_3_), 1.12 (s, 3H, CH_3_), 2.23 (d, 1H, *J* = 6.8 Hz), 2.28 (d, 1H, *J* = 6.6 Hz), 2.45 (d, 1H, *J* = 7.2 Hz), 2.42 (d, 1H, *J* = 7.2 Hz), 4.85 (s, 1H), 6.89–7.68 (m, 8H, Ar-H), 8.49 (s, 2H, NH_2_); ESI-MS: *m*/*z* 421 [M + H]^+^.

**2-Amino-3-(benzo[*d*]thiazol-2-yl)-4-(4-methyoxyphenyl)-7,7-dimethyl-5-carbonyl-5,6,7,8-4*H-*benzo[** ***b*]pyran** (**4i**): mp 176–178 °C. IR (KBr): 3398, 1668, 1654, 1623, 1474, 756, 664 cm^−1; 1^H NMR (200 MHz, DMSO-*d*_6_) (ppm): 0.89 (s, 3H, CH_3_), 1.05 (s, 3H, CH_3_), 2.12 (d, 1H, *J* = 6.8 Hz), 2.25 (d, 1H, *J* = 6.8 Hz), 2.46 (d, 1H, *J* = 7.2 Hz), 2.58 (d, 1H, *J* = 7.2 Hz), 3.69 (s, 3H, OCH_3_), 4.55 (s, 1H), 6.76–7.93 (m, 8H, Ar-H), 8.38 (s, 2H, NH_2_); ESI-MS: *m*/*z* 433 [M + H]^+^.

## 4. Conclusions

In summary, a simple, efficient and environmentally benign protocol for preparation of tetrahydrobenzo[*b*]pyrans was developed using novel basic, biodegradable, and cost-effective ionic liquid [Ch][OH] as catalyst in aqueous solution. Compared with the traditional imidazole derived ionic liquids, [Ch][OH] not only produced comparative or better results in terms of reaction rate and product yield but also is biodegradable, cheap and can be reused six times without significant loss of its catalytic efficiency. The applications of the novel ChCl-derived ionic liquids for other organic transformations are currently being investigated in our lab.

## Supplementary Information



## Figures and Tables

**Table 1.** Optimization of reaction conditions for the synthesis of tetrahydrobenzo[*b*]pyran a.

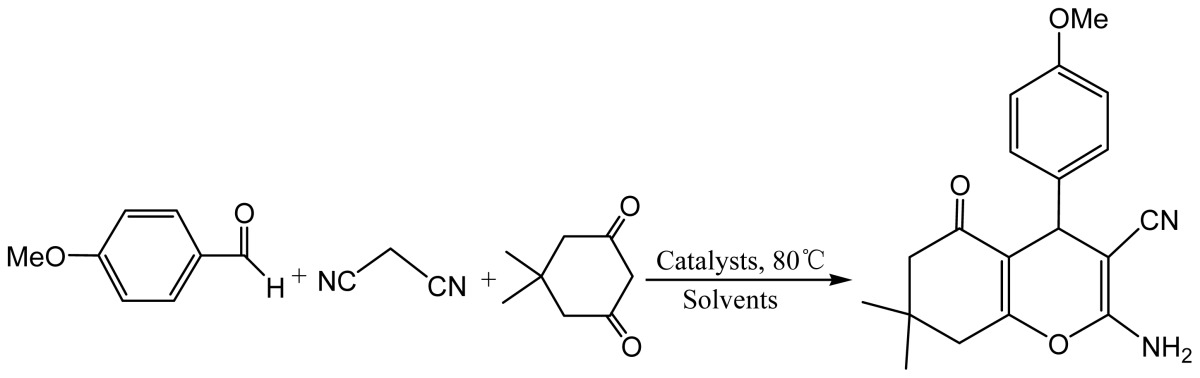					

Entry	Ionic Liquids	Mol %	Solvents (10 mL)	Time (h)	Yield (%) b
1	[bmim]BF_4_	20	Water	5	17
2	[bmim]PF_6_	20	Water	5	8
3	[DBU][Ac]	20	Water	2	31
4	[DBU][Lac]	20	Water	2	28
5	[Ch][Ac]	20	Water	3	52
6	[Ch][Pr]	20	Water	3	47
7	[Ch][Bu]	20	Water	3	55
8	[Ch][Lac]	20	Water	3	55
9	[Ch][OTf]	20	Water	3	38
10	[Ch][OH]	20	Water	2	95
11	[bmim]OH	20	Water	3	86
12	[Ch][OH]	10	Water	2	96
13	[Ch][OH]	5	Water	5	83
14	[Ch][OH]	30	Water	2	95
15	[Ch][OH]	50	Water	2	96
16 c	[Ch][OH]	10	CH_2_Cl_2_	5	Trace
17 c	[Ch][OH]	10	THF	3	Trace
18 c	[Ch][OH]	10	Ethanol	2	82
19 c	[Ch][OH]	10	Methanol	2	68

a All reactions were carried out as follows: the mole ratio of *p*-anisaldehyde: malononitrile: dimedone was 1:1:1 and the reaction temperature was 80 °C;

b Isolated yields;

c The reactions were conducted in the presence of reflux conditions.

**Table 2.** Synthesis of pyran derivatives via condensation of aromatic aldehydes, malononitrile and dimedone using [Ch][OH] as catalyst in water.

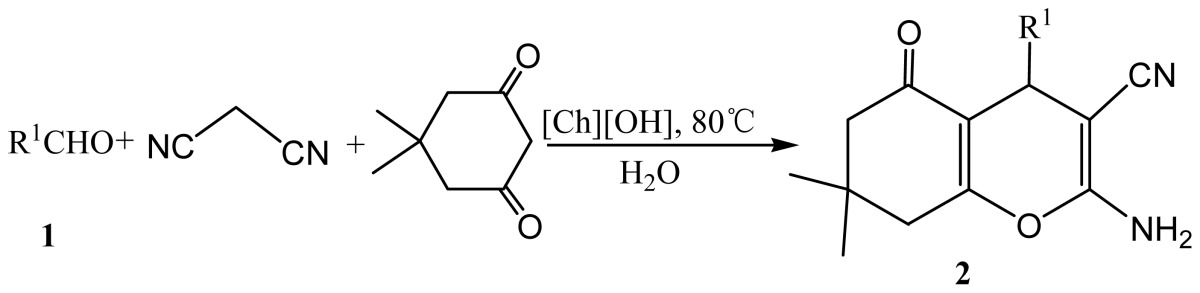						

Entry	R^1^	Time (h)	Products	Yields (%) a	*M*p (°C) Measured	*M*p (°C) References
1	C_6_H_5_	1	2a	92	232–234	234–235 [57]
	4-NO_2_-C_6_H_4_	30 min	2b	88	176–177	177–178 [27]
2	3-NO_2_-C_6_H_4_	20 min	2c	95	213–214	213–215 [27]
3	4-Cl-C_6_H_4_	1	2d	86	212–214	209–210 [19]
4	4-F-C_6_H_4_	10 min	2e	90	198–200	200 [28]
5	4-Br-C_6_H_4_	1	2f	91	165–168	169–170[30]
6	4-CN-C_6_H_4_	30 min	2g	90	227–229	224–226 [18]
7	4-OH-C_6_H_4_	2	2h	86	206–298	207–209 [28]
8	4-MeO-C_6_H_4_	3	2i	95	198–200	199–200 [28]
9	4-N(Me)_2_-C_6_H_4_	5	2j	98	216–218	217–218 [57]
10	3,4-2(MeO)-C_6_H_3_	3	2k	92	184–185	182–184 [14]
11 c	3-Pyridine	10	2l	0	-	-
12	2-Furyl	3	2m	82	223–226	222–224 [57]
13 c	2-Naphthyl	10	2n	0	-	-
14	2-Thienyl	1	2o	85	210–212	210–212 [18]
15 b	C_6_H_5_-CH=CH-	5	2p	68	205–207	208–210 [27]
16 b	CH_3_CH_2_	3	2q	85	190–192	190–194 [22]
17 b	CH_3_CH_2_CH_2_	3	2r	79	192–194	192–193 [58]

a Isolated yields;

b 20 Mol % amount of [Ch][OH] and reaction temperature of 100 °C were required;

c Only Knoevenagel condensation products were detected.
