# Supplementary material for: An Environmentally Benign Protocol for Aqueous Synthesis of Tetrahydrobenzo[b]Pyrans Catalyzed by Cost-Effective Ionic Liquid
Source: Int J Mol Sci. 2014 Apr 22;15(4):6897–909. doi: 10.3390/ijms15046897 (PMC4013668; doi:10.3390/ijms15046897)
Supplement: Supplementary file 1 [file ijms-15-06897-s001.pdf]

Supplementary Information

1. The pH Values of the Ionic Liquids

The aqueous solution of ChCl based ionic liquids (0.5 mmol/mL) were conducted at the Mettler Toledo pH meter (FE20) at 25 °C. The pH values are listed in Table S1.

Table S1. pH values investigations.

ILs	[Ch][OH]	[Ch][Ac]	[Ch][Pr]	[Ch][Bu]	[Ch][OTf]	[Ch][Lac]
pH	12.85	7.49	7.71	7.85	7.23	7.09

2. All Spectrums of the Choline Hydroxide Based Ionic Liquids

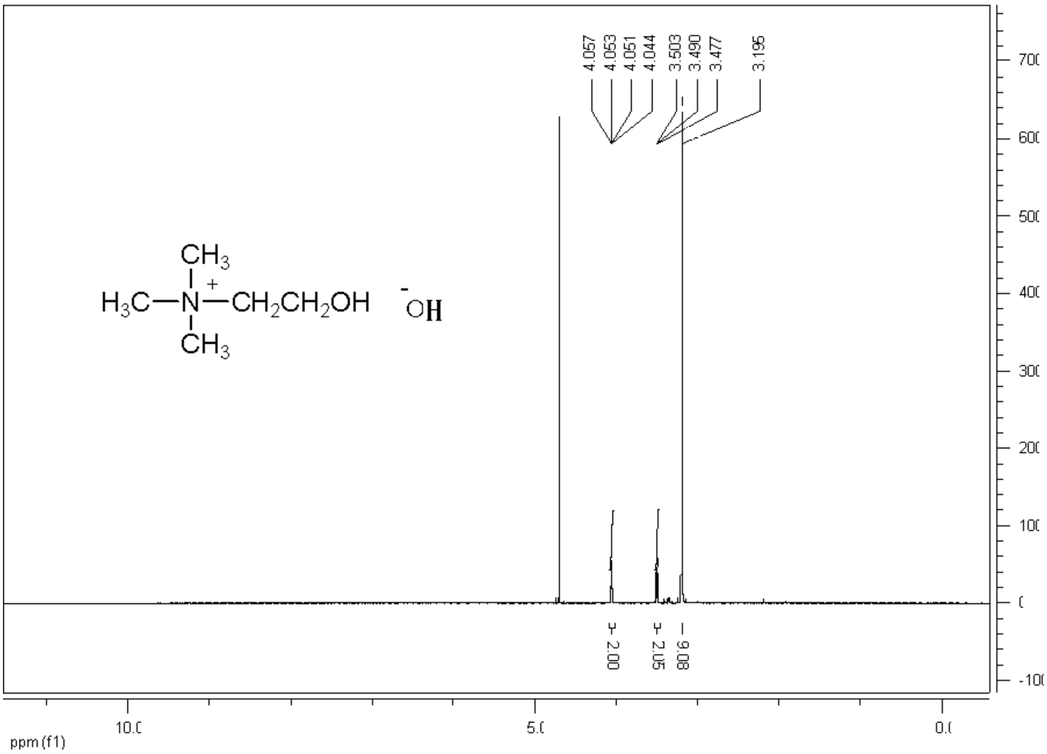

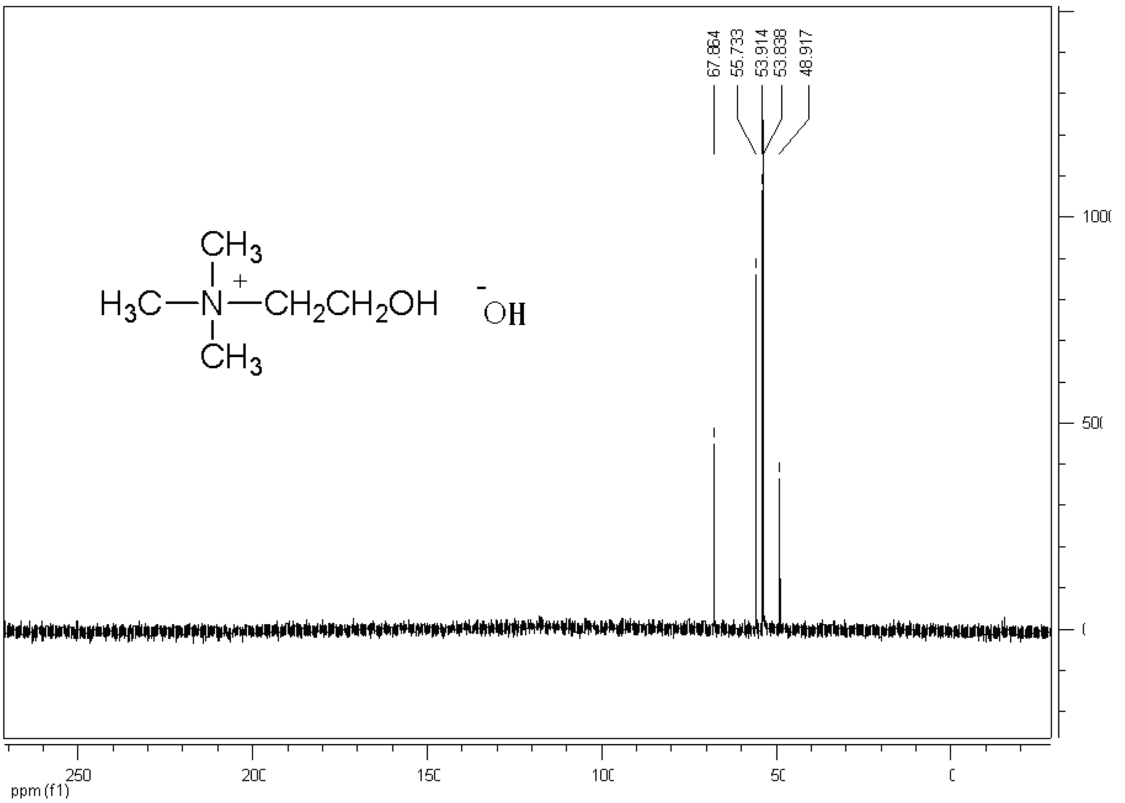
67.864
55.733
53.914
53.838
48.917
CH_3
$H_3C—N^+—CH_2CH_2OH$ ^-OH
CH_3
250
ppm (f1)

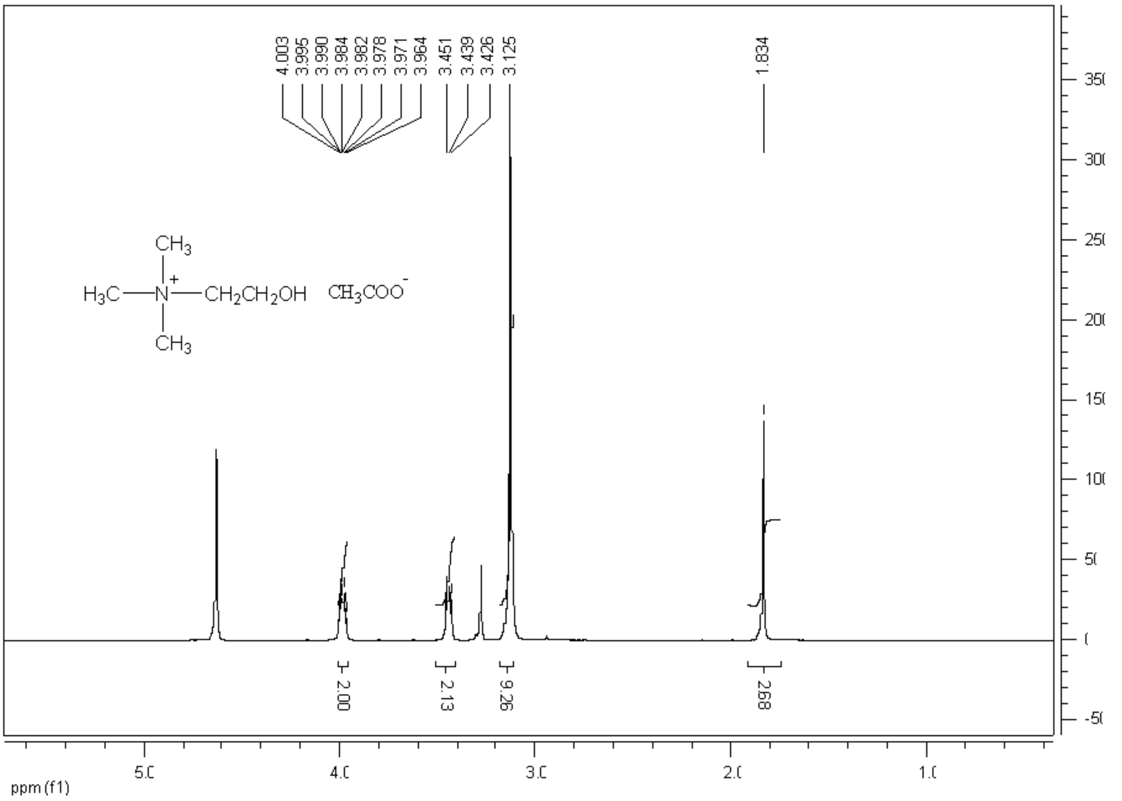
4.003
3.995
3.990
3.984
3.982
3.978
3.971
3.964
3.451
3.439
3.426
3.125
1.834
CH_3
$H_3C—N^+—CH_2CH_2OH$ CH_3COO^-
CH_3
2.00
2.13
9.26
2.68
ppm (f1)

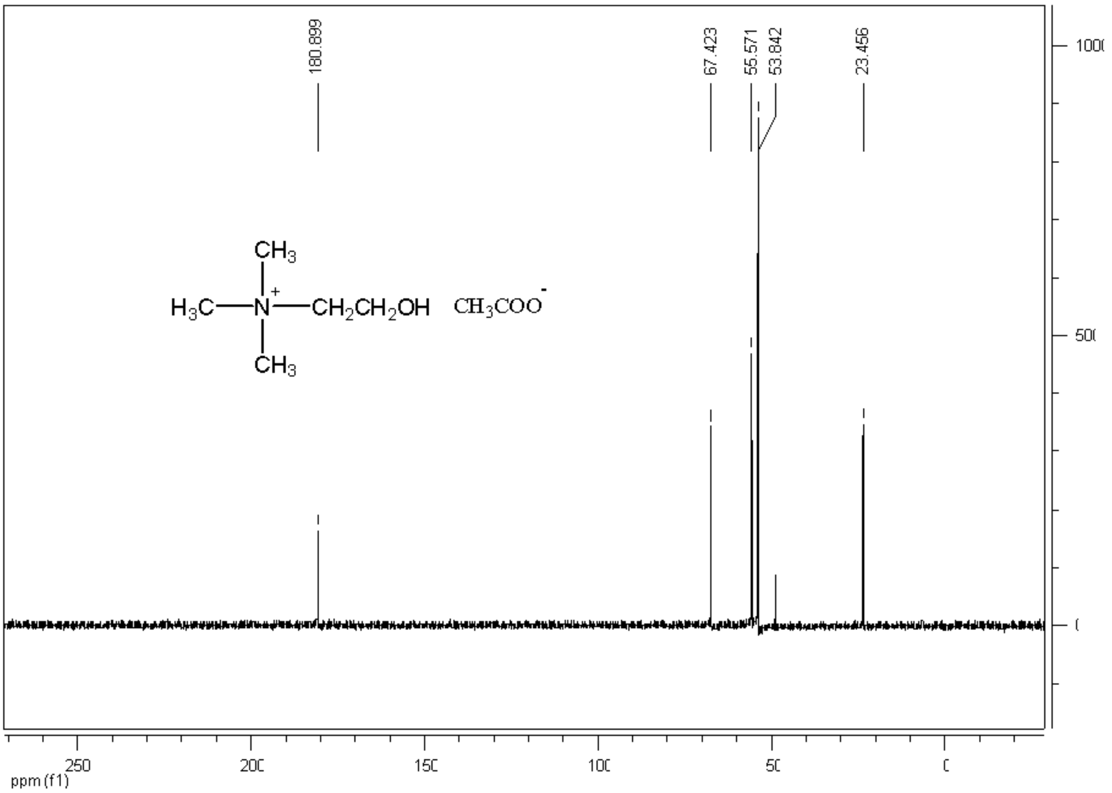
180.899
67.423
55.571
53.842
23.456
CH3
H3C—N+—CH2CH2OH CH3COO-
CH3
250
ppm (f1)

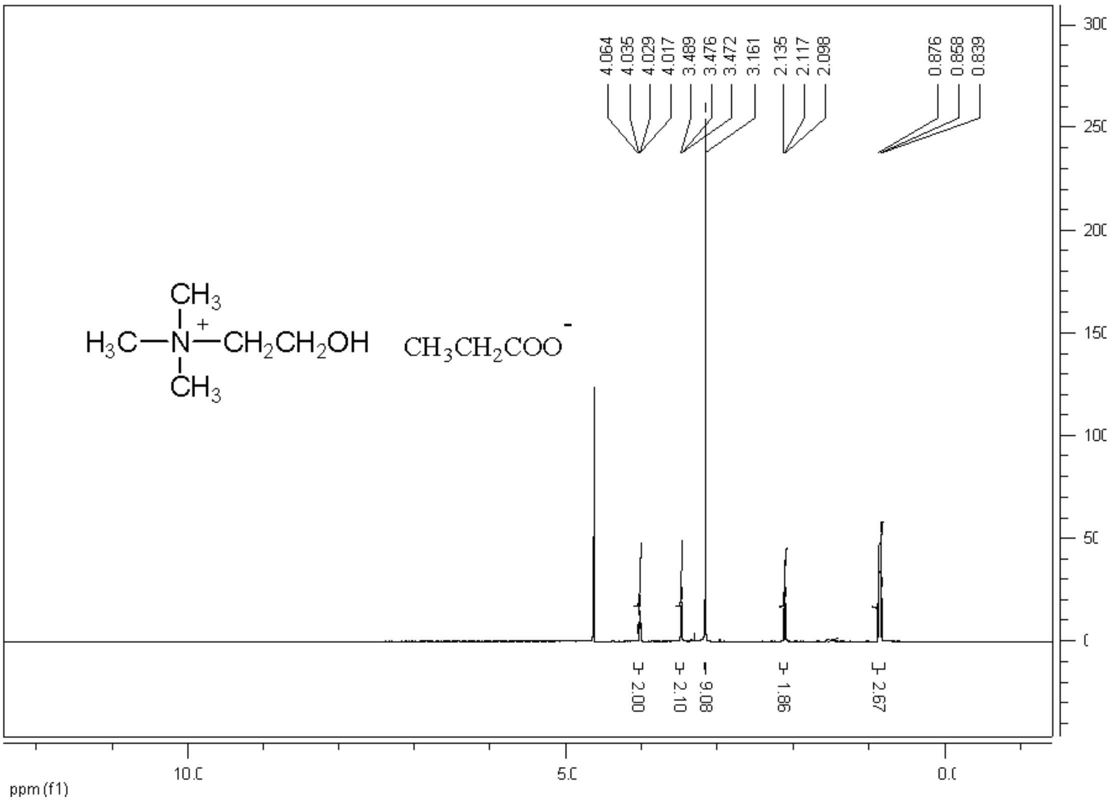
4.064
4.035
4.029
4.017
3.489
3.476
3.472
3.161
2.135
2.117
2.098
0.876
0.858
0.839
CH3
H3C—N+—CH2CH2OH CH3CH2COO-
CH3
2.00
2.10
9.08
1.86
2.67
ppm (f1)

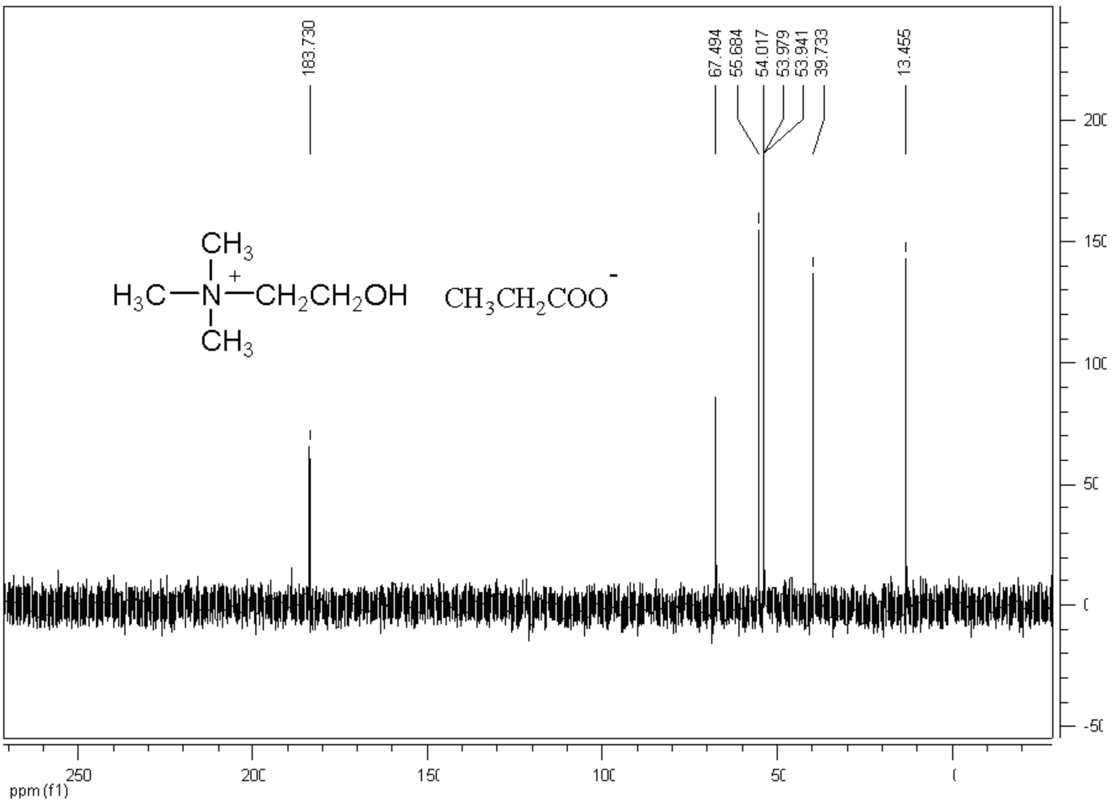
183.730
67.494
55.684
54.017
53.979
53.941
39.733
13.455
CH3
H3C—N+—CH2CH2OH CH3CH2COO-
CH3
250
200
150
100
50
0
ppm (f1)

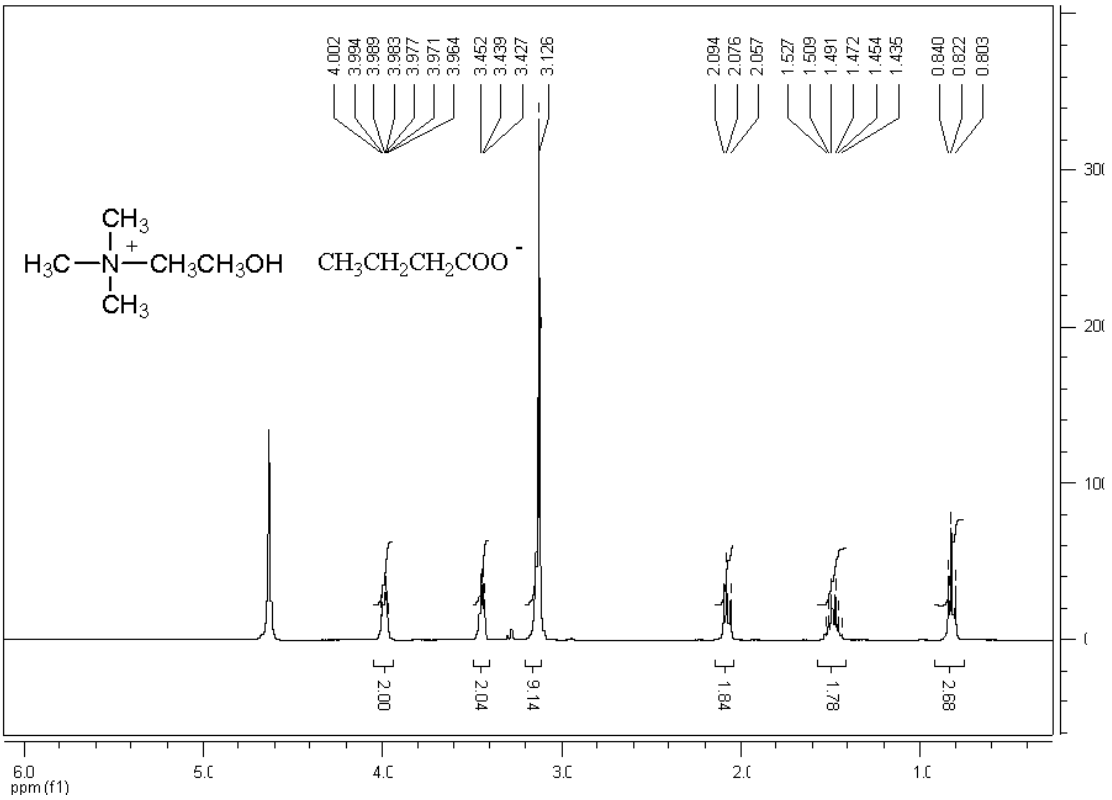
4.002
3.994
3.989
3.983
3.977
3.971
3.964
3.452
3.439
3.427
3.126
2.094
2.076
2.057
1.527
1.509
1.491
1.472
1.454
1.435
0.840
0.822
0.803
CH3
H3C—N+—CH3CH3OH CH3CH2CH2COO-
CH3
2.00
2.04
9.14
1.84
1.78
2.68
6.0
5.0
4.0
3.0
2.0
1.0
ppm (f1)

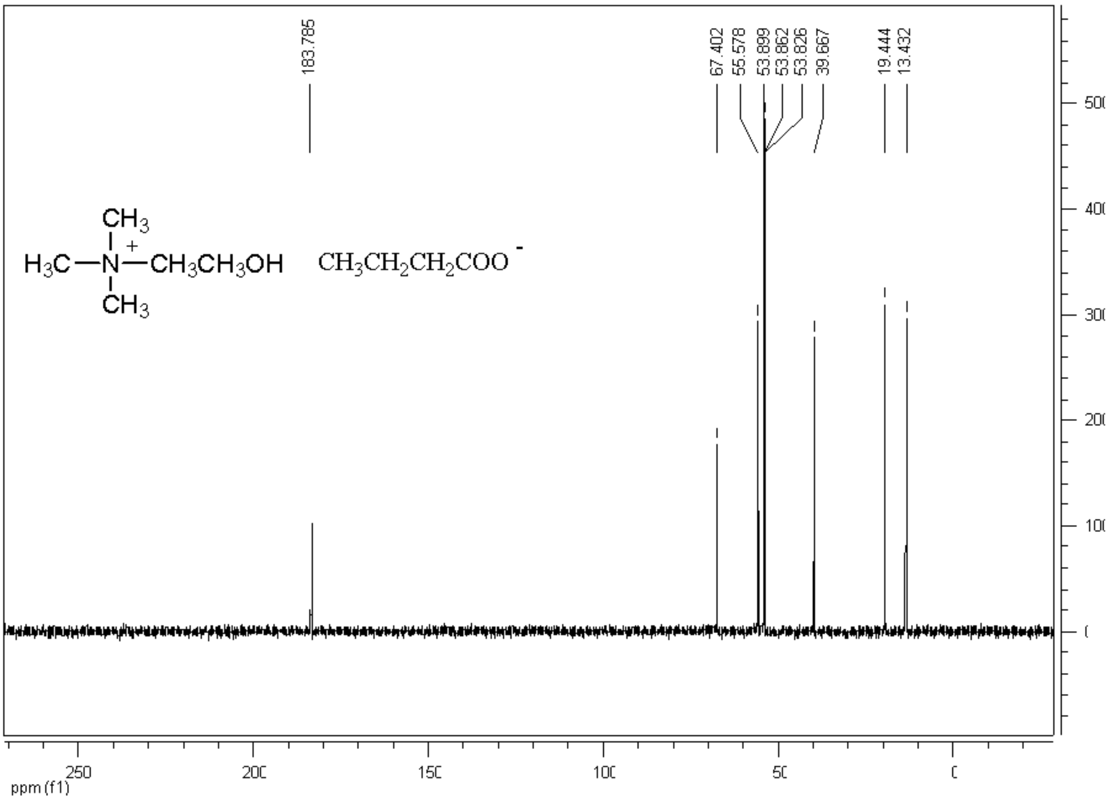
183.785
67.402
55.578
53.899
53.862
53.826
39.667
19.444
13.432
$H_3C-N^+(CH_3)_2-CH_3CH_3OH$
$CH_3CH_2CH_2COO^-$
250
200
150
100
50
0
ppm (f1)

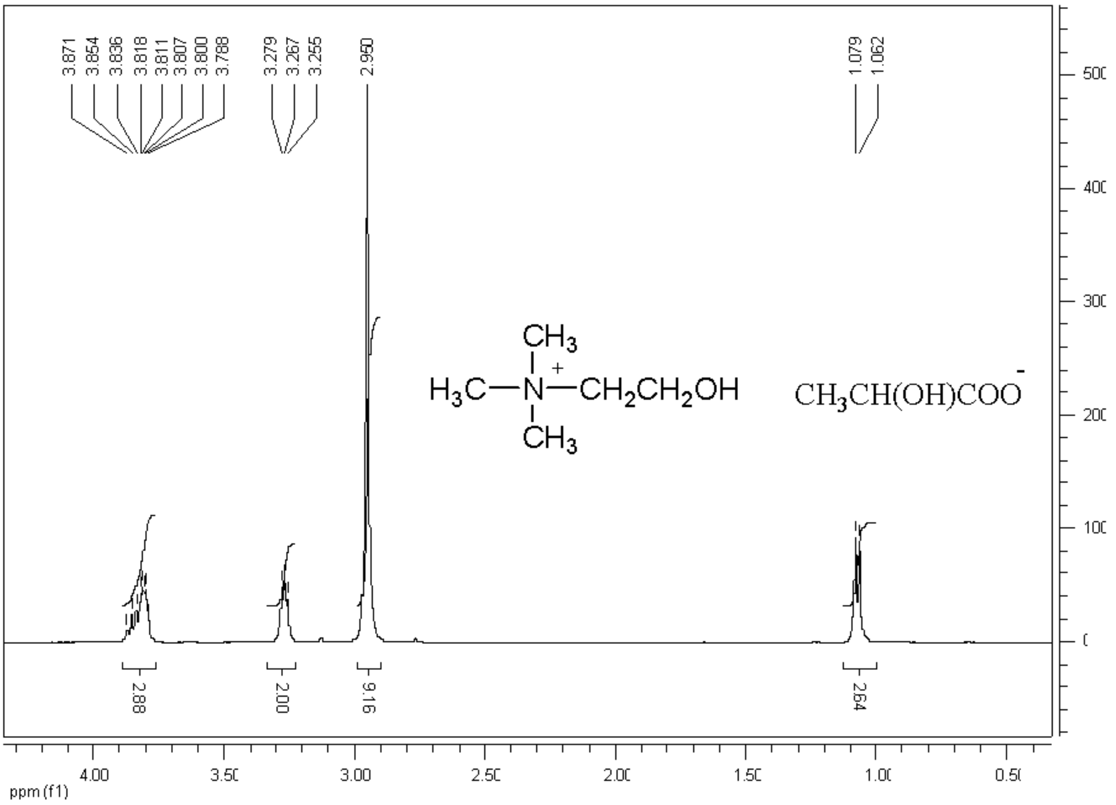
3.871
3.854
3.836
3.818
3.811
3.807
3.800
3.788
3.279
3.267
3.255
2.950
1.079
1.062
$H_3C-N^+(CH_3)_2-CH_2CH_2OH$
$CH_3CH(OH)COO^-$
2.88
2.00
9.16
2.64
4.00
3.50
3.00
2.50
2.00
1.50
1.00
0.50
ppm (f1)

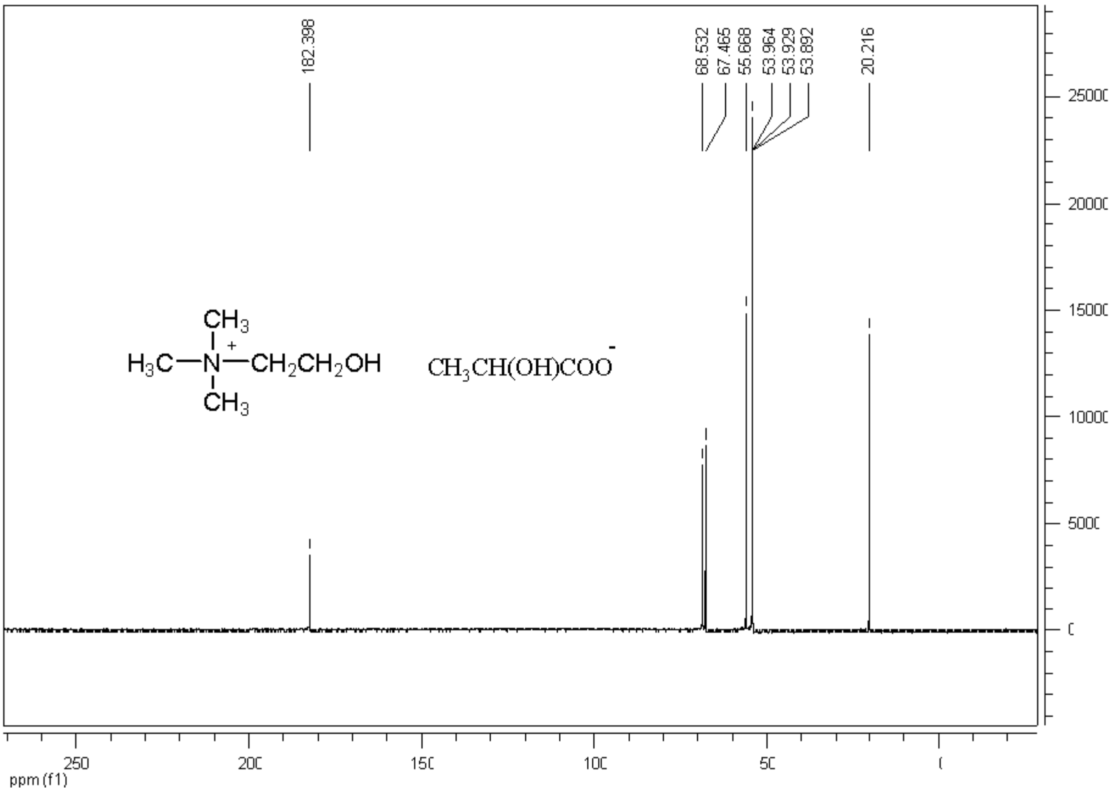
182.398
68.532
67.465
55.668
53.964
53.929
53.892
20.216
CH3
H3C—N+—CH2CH2OH
CH3
CH3CH(OH)COO-
25000
20000
15000
10000
5000
0
250
200
150
100
50
0
ppm (f1)

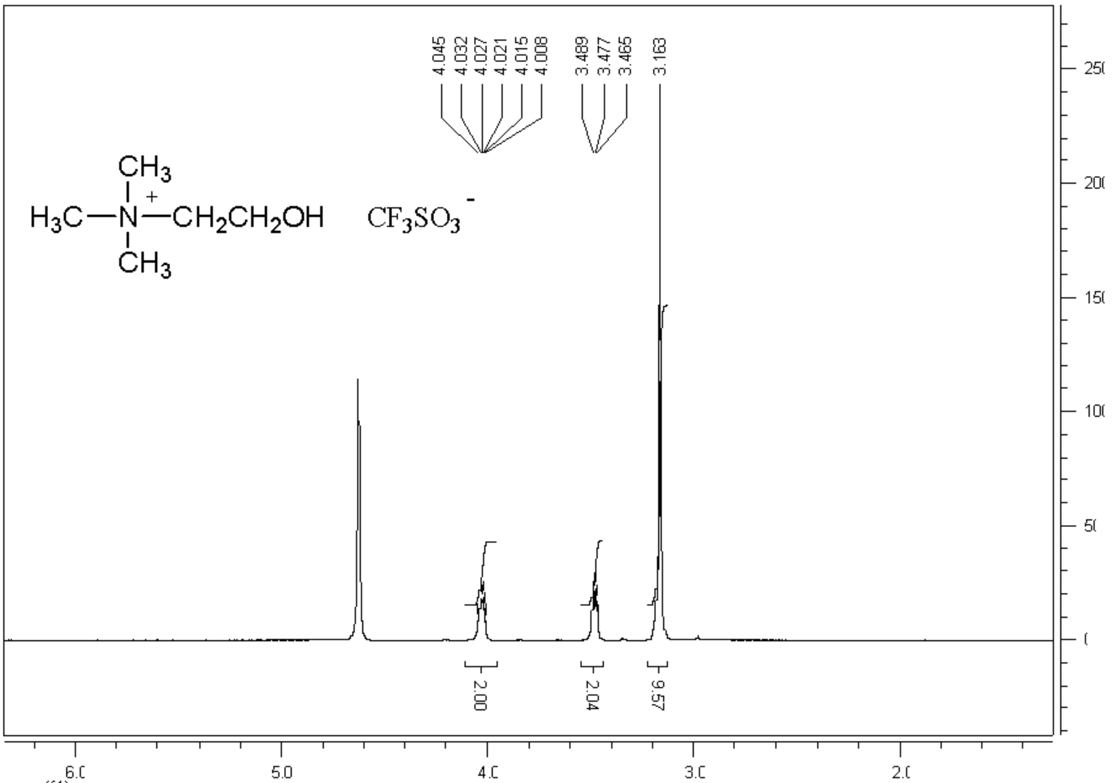
4.045
4.032
4.027
4.021
4.015
4.008
3.489
3.477
3.465
3.163
CH3
H3C—N+—CH2CH2OH
CH3
CF3SO3-
2.00
2.04
9.57
6.0
5.0
4.0
3.0
2.0
ppm (f1)

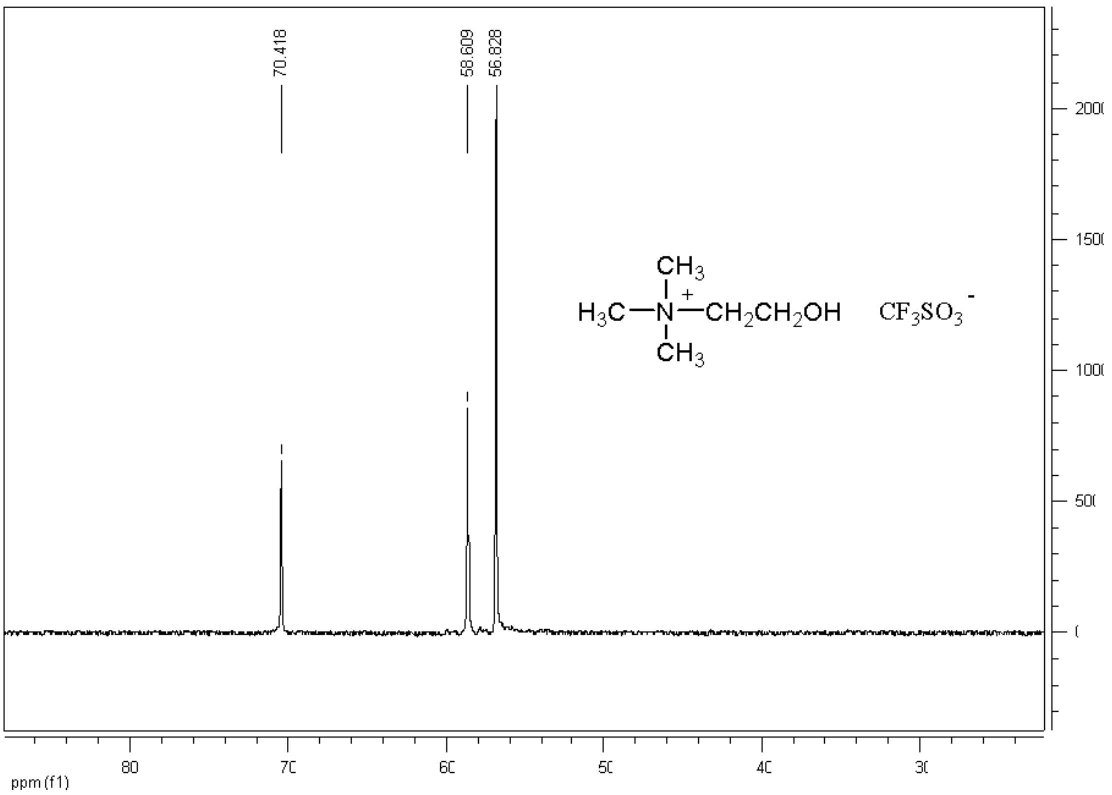

3. The Comparison of Two Carbon Spectrums to Confirm the Existence of Hydrogen Bond between Aromatic Aldehyde and Hydroxyl Group at the Side Chain of [Ch][OH]

Figure S1. Carbon spectrum of benzaldehyde in DMSO.

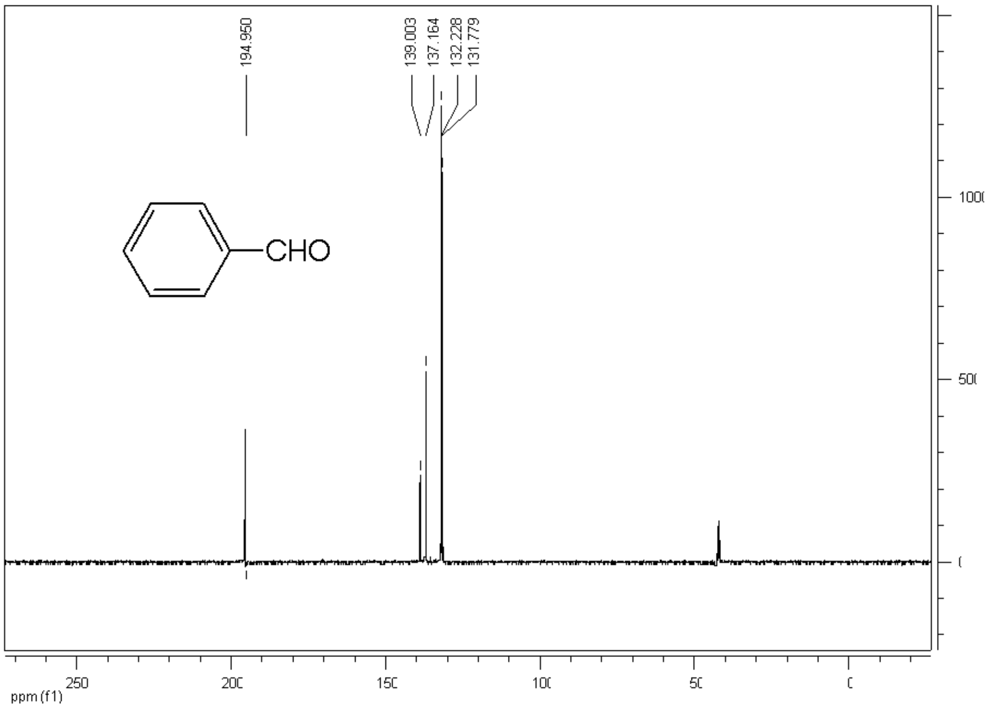

Figure S2. Carbon spectrum of the mixture of benzaldehyde and [Ch][OH] in DMSO. It can be observed from Figures 1 and 2 that the chemical shift is changed from 194.99 to 171.79 when equiv [Ch][OH] is added to the benzaldehyde, which shows the existence of the hydrogen bond interaction between carboxyl group of benzaldehyde and hydroxyl group of [Ch][OH].

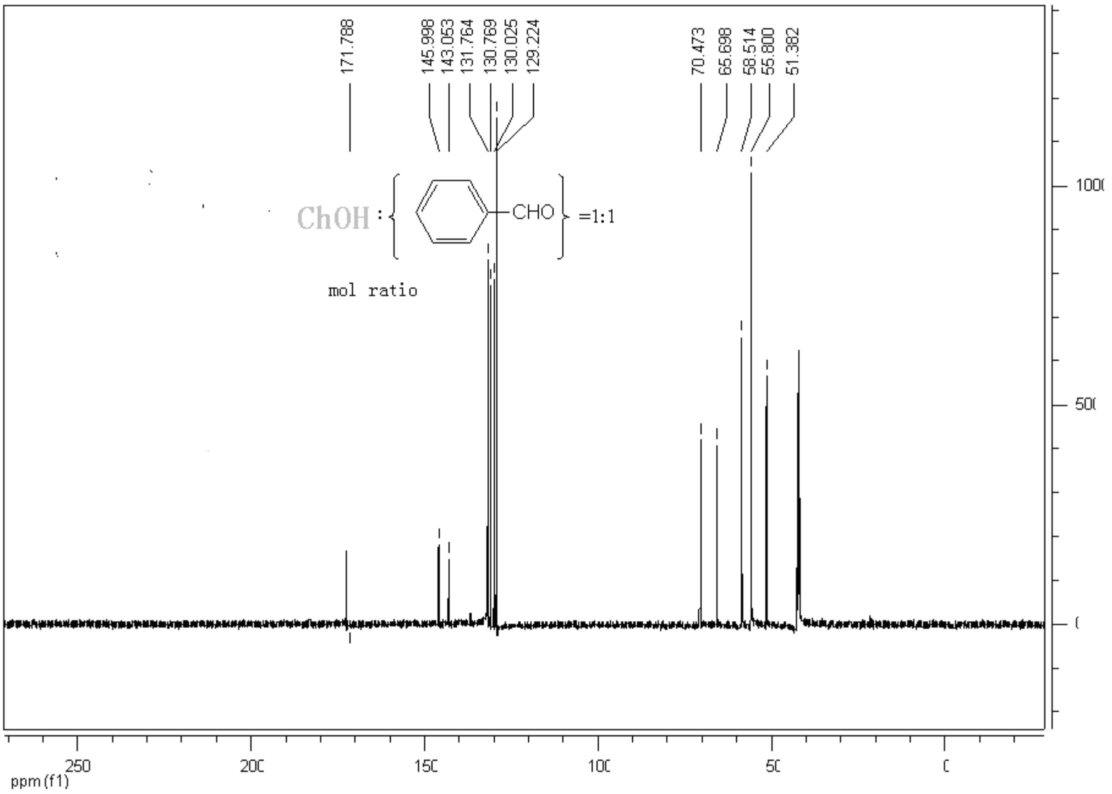

© 2014 by the authors; licensee MDPI, Basel, Switzerland. This article is an open access article distributed under the terms and conditions of the Creative Commons Attribution license (http://creativecommons.org/licenses/by/3.0/).
